# Parity and the Resolution of Value Conflicts in Design

**DOI:** 10.1007/s11948-022-00375-4

**Published:** 2022-04-13

**Authors:** Atay Kozlovski

**Affiliations:** Ethics Center, Philosophy Department, University of Zürich, Zürich, Switzerland

**Keywords:** Value sensitive design (VSD), Parity, Energy systems, Ruth Chang, Value conflict, Responsible innovation

## Abstract

Recent developments in theories for responsible innovation have focused on the importance of actively accounting for values in our technological designs. Leading among these theories is that of Value Sensitive Design (VSD) which attempts to guide the design process on the basis of evaluative analysis. However, values often come into conflict and VSD has been criticized for not providing a proper method to resolve such inevitable conflicts. This paper examines three such methods and argues that although each has its merits, they all fail to account for a common source of value conflicts known as value incommensurability. Drawing on literature from the field of axiology, this paper argues that by incorporating the evaluative relation of ‘parity’ each of these three methods, and the VSD framework in general, will be able to properly understand the relation which holds between conflicting design options stemming from the incommensurable of values and be able to guide designers in making rational decision in the face of such conflicts.

## Introduction

In recent years the field of responsible innovation has produced a variety of theories which focus on how to ensure that human values are implemented into technological designs. Leading among these theories is that of Value Sensitive Design (VSD) which proposes a tripartite investigative method—conceptual, empirical, and technical—for identifying relevant values that should be accounted for throughout the design life cycle. Over the last 25 years VSD has grown in popularity and has been applied in a variety of technological domains such as energy systems, healthcare technology, AI, and others (Winkler & Spiekermann, 2018). Through a combination of empirical studies, philosophical investigations, and technical analyses, the VSD framework generates a holistic evaluation which accounts not only for the interests and values of the design team, but also for that of a variety of stakeholders that are likely to be impacted by the technology being assessed.

This aim towards a holistic evaluation, an evaluation which accounts for a variety of values and interests, generates a practical and theoretical difficulty in the form of conflicting values.^1^ Consider for instance the following case which I will refer to throughout the paper: in their analysis on the design of nuclear reactor technology, Taebi and Kloosterman present the following evaluation of three reactors along four different evaluative dimensions: (Table 1).

**Table 1:** Assessing three nuclear reactor models along four evaluative dimensions. *Source*: Taebi, B., & Kloosterman, J. L. 2015, 827

	HTR-PM	GFR	MSR
Safety	+ +	−	+
Security		− −	−
Sustainablitiy (durability)	−	+	
Economic viability	+	0	−

This is an internal comparison based on each criterion or value (aassigning + and − as a means of internal comparison based on value does not imply that we can quantitatively compare these values. In other words, we cannot sum up the + and − for each reactor to see which one scores best)

From the table we see that there is no one model which scores highest along all four criteria. This lack of an overall best model entails that a value trade-off is inevitable in that any choice of model will be based on our prioritising one value over another. Although this issue has been repeatedly discussed in the VSD literature, there is still no consensus as to what method ought to be adopted in order to resolve such conflicts in design. In this paper I will look at three proposed methods and argue that although each has its merits, they all eventually fail to properly account for an important source of value conflicts which is the incommensurability of those values. Drawing on literature from the fields of axiology and normative ethics, I will argue that each particular method, and the VSD framework as a whole, would benefit from incorporating the evaluative relation of ‘parity’ as a way of understanding how incommensurable alternative evaluatively relate and what to do when such values come into conflict.

The paper is structured as follows: The section “Conflicting Values in Design” gives an overview of the VSD methodology and discusses the identification of value conflicts in design. In the next section, “Existing Methods for Resolving Value Conflicts in Design”, I examine three existing methods within the VSD literature. The section “Value Incommensurability” then introduces the phenomenon of value incommensurability and shows how it poses significant problems for each of three methods from section “Existing Methods for Resolving Value Conflicts in Design”. In the final section, “Parity”, I discuss the notion of parity and its relation to rational decision making. I then go on to show how parity can complement and improve each of three methods for value conflict resolution and the VSD framework in general.

## Conflicting Values in Design

### Value Sensitive Design

Originally developed by Batya Friedman and Peter Kahn, VSD is described as “a theoretically grounded approach to the design of technology that accounts for human values in a principled and comprehensive manner throughout the design process” (Friedman et al., 2013. p. 56). The aim of VSD is to equip designers with the necessary tools and skills for identifying and embedding relevant values into their product. To succeed in this endeavor VSD proposes a tripartite research method aimed at eliciting relevant values for the design. This three part methodology consists of conceptual, empirical, and technical investigations into the relevant product. Although each investigation has its own primary focus, they should not be perceived as separate and unrelated stages. Rather, these investigations are meant to complement one another and may be repeated several times as new data is obtained. The conceptual investigation focuses on two aspects: the identification of direct and indirect stakeholders, and the philosophical analysis of relevant values. The empirical investigation performs a variety of field studies such as interviewing experts or stakeholders, conducting surveys, testing prototypes and so on. Finally, the technical investigation informs designers as to the state of the art of the existing technology, possible innovations, material or technical limitations, and which values are promoted by the different technological features.

Before moving on to the issue of conflicting values, it will be helpful to address a few preliminary general issues regarding VSD. To begin, it may strike some as an odd idea that values can be embedded in a technological product. This of course does not mean that the value of justice is somehow made physical and inserted into a computer program. Rather, consider how a social policy such as ‘unemployment benefits’ does not only provide money for those who have lost their job but also promotes certain values such as egalitarianism, security, well-being and so on. In similar fashion, the fact that we have developed an affordable high speed internet technology can potentially promote values such as literacy, accessibility, reduced social inequality and more. By accounting for these values in the design process we have ‘embedded’ them in our new product.^2^

Another important issue to stress here at the outset is that although VSD was first applied in the field of HCI (Friedman & Kahn, 2003) and is framed in terms of supporting the design process of a technological product, much of the discussion in this paper will focus on decision making and decision makers as opposed to engineers or designers. This shift is important as it stresses the fact that VSD is most useful insofar as we accept the idea that it is the decisions that are made throughout the design process which allow us to account for the values we wish to promote. In this respect VSD could theoretically be used not only for an analysis of technological design but also to guide policy decisions, institutional reform, and even personal life choices.

Finally, there are two common critiques of VSD which will be partially addressed in this paper. First, VSD is said to lack a complementary ethical theory which would support value elicitation and the resolution of value conflicts (Manders-Huit, 2011, Van den Hoven et al., 2012). While this paper will not attempt to endorse any specific ethical theory, it will address this issue by engaging in the debate regarding substantive vs procedural ethical frameworks.^3^ This distinction focuses on whether the ethical framework picks out specific values in advance or whether it emphasizes the procedure by which values are elicited and ranked.

Second, VSD places great emphasis on identifying relevant stakeholders and accounting for their values in the design process. The idea of stakeholders was incorporated into VSD as an attempt to answer the question of “whose values are to be taken into account” (Friedman & Hendry, 2019, 35) in the design. In its widest understanding stakeholders are “those who are or will be significantly implicated by the technology” (Ibid). Four categories of stakeholders are mentioned in the VSD literature: project sponsors, designers, direct stakeholders, and indirect stakeholders. The distinction between direct and indirect stakeholders revolves around whether the stakeholders engage directly with the technology or are merely affected by its existence. The critique of VSD in this respect relates to the fact that the framework does not provide designers with a clear procedure for identifying stakeholders and does not specify how to deal with contrasting values from distinct stakeholder groups. This paper will not address the issue of how to identify stakeholders but by discussing the issue of value conflicts I hope to shed some light on the latter of these two critiques.

### Value Elicitation and Conflict

According to Friedeman the concept of ‘values’ refers to “what a person or group of people consider important in life” (Friedman et al., 2013. p. 56). As such, VSD recognizes not only abstract values such as justice, equality, and autonomy, but also more concrete values such as protection from flooding, parental supervision of teenagers, or social support for weight loss. Along these lines, the three VSD investigations—conceptual, empirical, and technical—constitute the different methods by which designers can identify values that need to be taken into account in their design and see whether any of these values come into conflict.

A good example of how VSD identifies value conflicts can be seen in Mok and Hyysalo’s (2017) VSD analysis of a project to implement solar energy technology in a cultural heritage site at Aalto University in Finland. In their conceptual investigation they identified four key values for their design: cultural heritage preservation, campus prestige and image, ecological modernization, economic cost and space viability. From their conceptual analysis Mok and Hyysalo predicted that the values of *cultural heritage preservation* and *ecological modernization* “could potentially conflict with one another irresolvably” (Mok and Hyysalo, 7). To further examine this hypothesis Mok and Hyssalo tested several prototypes as part of their empirical investigation and succeeded in identifying a potentially acceptable compromise in what they call a model with ‘subtle visibility’ (Ibid, 15).

This example along with the nuclear reactor case discussed in the introduction show that there are a variety of ways in which values can come into conflict. While in some design cases two values will appear to be mutually exclusive, in other situations the available alternatives may promote all the relevant values but to different degrees. The various ways in which values can come into conflict have also engendered different strategies for dealing with such cases. Friedman discusses three approaches which have been used in the VSD literature: design trade-offs, value conflicts, and value tensions (Friedman & Hendry, 2019 44–45). These three approaches represent different ways in which a design team can approach a potential conflict in values. In a design trade-off we envision a situation in which one value is necessarily promoted at the expense of another. In contrast, in the value-conflicts and the value-tensions framings the designers seek solutions which simultaneously promote all the different relevant values.

For instance, consider a potential conflict between the values of security and accessibility in the design of a security system for medical records of hospital patients. While we need to ensure that these records remain confidential, we also want to allow doctors and caretakers to have quick and easy access to important information (Hedström et al., 2011). If we approach this design situation from a design trade-off perspective we may come to believe that any solution we choose would eventually either maximize information security or accessibility and that our main task is to determine which is more important. In contrast, Friedman believes that if we begin the design process with a value conflict or tension outlook, the set of acceptable design alternatives may be wider in that we could accept solutions which “do not necessarily optimize one value at the expense of the other” or “balance each value in relation to the others” (Friedman & Hendry, 2019 44–45). Although the specific situation will often determine which is the relevant framing, Friedman argues that the value tension outlook should be adopted when possible as it promotes the broadest design thinking.

## Existing Methods for Resolving Value Conflicts in Design

In the previous section I discussed the issue of value conflicts in VSD and the different theoretical framings that a designer can adopt when attempting to resolve these cases. However, it is important to notice that regardless of how we choose to frame the issue of values in conflict, eventually some kind of method will be needed for choosing between the alternative ways to proceed with the design. In this section I briefly examine three existing methods which have been developed in order to guide design choices in the face of value conflicts. I then go on to argue that although helpful in many ways, each of these methods struggles to provide proper guidance in the face of a phenomenon called value incommensurability. The idea of value incommensurability, which I discuss further in section “Value Incommensurability”, is that some values cannot be reduced to a single scale of measurement and as such pose a challenge to rational decision making when they come into conflict.

### ‘Value Dams and Flows’

The first method, developed by Miller et al., (2007) offers an empirically informed method based on stakeholder preference analysis to uncover potential value tensions in the design and then help guide designers to deal with necessary design trade-offs. This method suggests that designers pass out surveys to stakeholders in order to identify specific product features that would constitute either ‘value dams’ or ‘value flows’. “Value dams refer to technical features or organizational policies that are strongly opposed by even a small set of stakeholders” (Ibid, 284). That is, value dams are perceived as the potential value tensions which need to be addressed. In contrast, “Value Flows refer to technical features or organizational policies that, for value reasons, a large percentage of stakeholders would like to see included in the overall system” (Ibid). By identifying value dams and flows designers are then able to adjust their product so as to promote the flows and minimize or eliminate the dams. For instance, while applying their method to help guide the design of a code sharing platform, Miller et al. discuss the value tension between privacy and awareness (Ibid, 285). From their collected data they elicited the following relevant dams and flows:

- Privacy Dams—logging of both searches and queries of users in the system.
- Awareness flows—the system should report how often contributions were used and how the different posts were ranked by users.

From this Miller et al. concluded that in order “to mitigate the privacy-related value dams while still taking advantage of the awareness-related value flows, we determined not to log or report who searches or queries, but to log and report frequency of code use and implement content ranking” (Ibid).

### Substantive Ethical Frameworks

A second method for resolving value conflicts proposes to complement the VSD framework with a substantive ethical theory which could then guide designers in cases of value conflicts. This method was suggested by Manders-Huit (2011) and then developed in a variety of publications. For instance, van Wynsberghe (2013) discusses her ‘care centered value sensitive design’ (CCVSD) for use in the design of healthcare robots. Drawing on the care ethics literature, van Wynsberghe argues that all care values are subsumed into four moral elements which together form the normative portion of her framework: attentiveness, responsibility, competence, responsiveness (van Wynsberghe, 2013, 419). In addition to these basic values, the framework stresses the need to adjust any design to a variety of variables such as context (hospital, nursing home), practice (lifting, feeding..), actors involved, and type of robot (assistive or replacement) (Ibid, 420). The underlying assumption seems to be that the moral framework and the high level of contextualisation would then resolve any value conflict which could arise during the design process.

### Value Hierarchies, (Re)Specification, and Innovation

In a series of publications Van de Poel (2013, 2015, 2017) examines the relation that exists between values, norms, and design requirements, and develops his ‘Values Hierarchies’ method. Essentially this method shows how we can move from very abstract values such as justice or freedom, to less abstract norms which interpret these values, and finally to specific design requirements which can be derived from the norms. It is important to stress that unlike the previous substantive methods, this is more of a procedural account in that the relation between values, norms, and requirements is not of a deductive nature but can be open to dispute.

After the designer has completed the ‘Values Hierarchies’, if a conflict in values arises, Van de Poel then suggests two methods for resolving such cases: (re)specification and innovation. Specification refers to the interpretation which is given as we transition from one level of the hierarchy to the next. Accordingly, in the case of value conflicts respecification tells us to reconsider how we interpreted the higher level and whether a suitable alternative specification can be found such that the conflict is then eliminated. In contrast, innovation is a method which attempts to deal with value conflicts by “developing new, not yet existing options” (Van de poel, 2017, 64). Van de Poel suggests that both strategies should be used at different stages during a design process. Innovation will be most useful in the early stages of the design when we are still gathering all our data and developing the design alternatives. In contrast, respecification will come in handy in the later stages of the process when choices need to be made among the different alternatives (Ibid, 67).

An example of this strategy at work was given with respect to the development of biofuels. The identified conflict relates to intergenerational vs intra-generational requirements. Biofuels are more sustainable in terms of greenhouse gas emissions (intergenerational) but compete with food production driving food prices up (intra-generational). Using first the method of innovation, Van de Poel suggests that the value conflict would disappear if we could produce third generation biofuels made from bacteria and algae. Such an innovation would clearly settle the value conflict but at present these biofuels are still being researched. As such, we now need to employ the second method, respecification. At a first pass Van de Poel suggested the following specification:
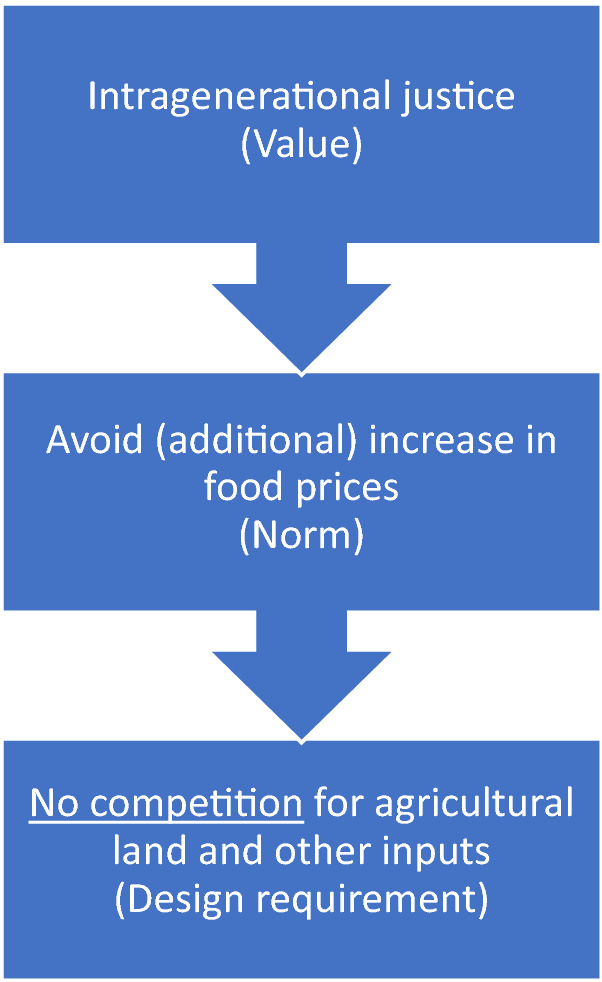


However, as first generation biofuels would directly compete with food production, this specification led to a value conflict. Van de Poel then suggests a respecification:
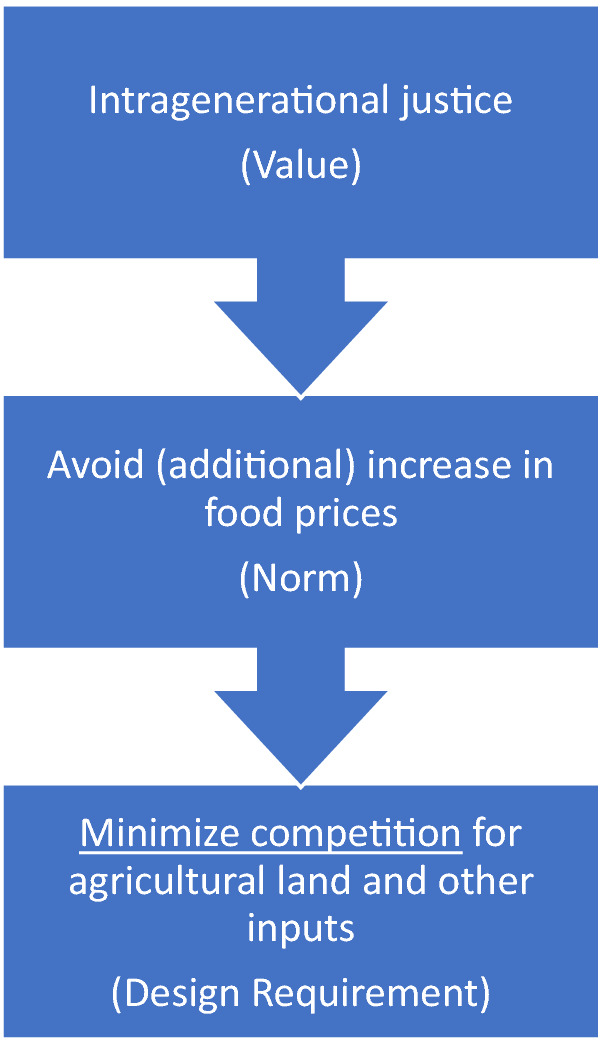


The respecification from ‘no competition’ to ‘minimize competition’ could resolve the conflict by guiding the design decision towards second generation biofuels which would still compete with food production but to a much lesser degree than first generation biofuels.

## Value Incommensurability

In this section I discuss the notion of value incommensurability and argue that, in their current formulation, this phenomenon poses a significant challenge to each of the methods discussed in the previous section. Formally, the notion of in/commensurability refers to the in/ability to evaluate items^4^ using a single unit of measurement or scale. For instance, if we were asked to evaluate ‘which of two trees is taller?’ we could simply measure both trees using the unit of ‘meters’ and then determine which is greater in this respect. As such, we say that the two trees are commensurate in terms of their height. In contrast, if we were asked to determine ‘which of two policy decisions is more just?’, we could not simply measure the level of ‘Justones’ in each policy decision and then answer in accordance. The value of justice lacks a unit of measurement and so the two policy decisions are incommensurable in terms of justice.

Some theorists have gone so far as to argue that there is no resolution for comparisons involving incommensurable alternatives claiming that incommensurability entails incomparability.^5^,^6^ However, this position does not align with the intuition that even if we cannot measure the amount of ‘Justones’ in two competing policy decisions we can still determine that one policy is more ‘just’ than another. Although there is no consensus on this matter in the axiological literature, one school of thought^7^ argues that value incommensurability generates a form of imprecision in our evaluations which leads us to conclude that in a comparison between incommensurable options X and Y, it can be the case that neither option is better than the other nor are they equally as good. That is, while between commensurable alternatives one option will always be either better, worse, or equal to the other, in comparisons between incommensurable alternatives we may conclude that none of the three standard relations (better, worse, and equal) holds in that specific case.

Most commonly our inability to determine which option is better will result from the fact that X will be better in some relevant respect, Y will be better in other relevant respects, but neither option will be best all-things-considered. Let’s see how this may happen in a revised version of the nuclear reactor example:

**Table:** 

	Security (Y)	Sustainability (Z)
HTR-PM	8Y	4Z
MSR	3Y	9Z

If we assume that the values of security and sustainability are incommensurable in this comparison this means that there exists no exchange rate between Ys and Zs. This in turn entails that MSR can be either better, worse, or equal to HTR-PM, or none of the above. If for instance it was determined that 3Y was an unacceptable level of security we could conclude that HTR-PM was the better option. Alternatively, if we needed to maximize sustainability at any cost then we would argue that MSR was the better choice. But sometimes we will not have any such side constraints and will be left only with the option of saying that MSR is the more sustainable option, HTR-PM is the more secure alternative, but neither is best overall. There is of course also the option that the two alternatives are equally good. This relation is mostly rejected via an argument known as the ‘small improvement argument’,^8^ but as a discussion of it will take us too far afield we can simply reject the equality option by clarifying that evaluative equality entails complete interchangeability of the alternatives. The qualitative difference between these two reactors (one very sustainable, the other very secure) makes it implausible for us to determine that these are exactly equally viable options. In the next section I will advocate that in cases like this in which none of three standard relations holds, we should accept that the options relate in terms of a fourth value relation called parity. Before that let us now look at how the phenomenon of value incommensurability challenges the three methods from the previous section.

Starting with ‘Value Dams and Flows’, this method essentially proposes that by deliberating with relevant stakeholders we can identify value conflicts (value dams) and eliminate them from the system. This method will be especially useful for very flexible technologies such as computer software. However, the general problem with this method is that it relies heavily on empirical data and runs the risk of leading designers to commit the naturalistic fallacy of presuming that the preferences of stakeholders are equivalent to the normative question of what ought to be done.^9^ In the next section I will argue that by acknowledging the possibility of value incommensurability and the relation of parity, we will be able to avoid this issue by identifying in which cases stakeholder preferences should be a determinate issue in the design.

As to the substantive ethical theory methods, value incommensurability poses a significant challenge to this method if the chosen theory is pluralistic in nature. Pluralistic theories, like the one proposed by van Wynsberghe, presuppose several fundamental values which cannot be reduced to any one ‘super value’ like in the case of utilitarianism or hedonism. In other words, such pluralistic theories essentially identify a set of incommensurable values which are supposed to help guide the design process. While this is very helpful for the VSD analysis, ironically it lays the groundwork for value conflicts like in the example of the nuclear reactors. That is, knowing which are the fundamental values of the ethical theory will not prevent those values themselves, or the bearers of those values, from coming into conflict. In such cases we will either need some predefined method for resolving such conflicts or a hierarchy among the different fundamental values. But as the latter option is ruled out by the very nature of the pluralistic theory, we are left only with the option of having some method for resolving conflicts between incommensurable alternatives, which is exactly the problem that is being examined here.

Lastly, Van de Poel’s method seems especially susceptible to the issue of value incommensurability. Recall that in constructing the ‘values hierarchies’ we need to specify how the value is translated into norms and then into design requirements. Van de Poel stresses that although not every kind of specification will be legitimate there are still a number of different legitimate specifications—“Specification is non-deductive which means that the initial norm can usually be specified in various ways” (Van de Poel, 2017, 63). Incommensurability seems to pose a challenge to this method in three different ways: First, the designers may not be able to identify which among two ‘acceptable’ specifications ought to be selected (neither is better, nor are they equally good). Second, the value based grounds on which we need to respecify in case of conflict may come across the same problem. Third, designers would not be able to justify a specific innovation path in the face of incommensurable options. In short, each of these three points emphasizes that in the face of conflicting and incommensurable alternatives Van de Poel’s method seems not to provide designers with sufficient guidance.

## Parity

This final section will discuss the evaluative relation of parity suggesting that it properly describes the relation that holds between incommensurable alternatives when we conclude that neither is better nor are they equally good. I will then go on to show how we make rational decisions in the face of parity and how by incorporating the relation of parity we can fill in the missing gaps in the three methods discussed in the previous section. I end this section with some suggestions as to how parity also adds some new layers to the VSD framework in general.

The evaluative relation of parity was developed by Ruth Chang (Chang, 2002) as a way to describe how incommensurable alternatives can evaluatively relate when neither is deemed to be better nor are the two equally as good. Although the existence of such a relation is still disputed,^10^ I will not engage here in this debate and will presuppose that parity can be defended. So what does it mean when we say that two alternatives are on-a-par (parity)? It means that we have sufficient reason to choose either on-a-par alternative but that each is favored by a different value which is relevant for the design and that the two options are qualitatively very different. With respect to our nuclear reactor example we could say that MSR and HTR-PM are on-a-par as the former is favored on grounds of sustainability, the latter is favored for reasons of security, and overall neither is better.

Once we have determined that we are dealing with on-a-par alternatives, we now need to discuss how rational decisions are made in such cases. In general, when making decisions based on an evaluative analysis there is a direct correlation between how we resolve a comparison in terms of which evaluative relation holds (better, worse, equal, or ‘on-a-par’) and what we ought to rationally do / choose in that case.^11^ When one option is judged to be all-things-considered better we have ‘most reason’ to choose that option and rationally ought to do so. If two options are deemed to be equally good we have sufficient reason to choose either option and rationally ought to randomly pick one of the two options. This is where the story ends in most theories of rational decision making. For instance, in many MCDM theories^12^ (multi criteria decision making) or in different social psychology theories^13^ we need to assign a weight variable to each value in order to always arrive at the conclusion that either one option is better or the two are equally good. However, in light of our discussion of incommensurability we can see that such theories ignore the existence of incommensurable values and presuppose that all values are fully commensurable. Parity proposes a way for us to accept the notion of incommensurability and act in accordance rather than trying to ‘fit a square peg into a round hole’.

According to Chang, cases of parity represent instances in which our reasons have ‘run out’. That is, in such instances the evaluative facts and the reasons that we have which normally determine which option ought to be rationally chosen have failed to settle the matter for us. This failure is not of a contingent nature but intrinsic to the comparison at hand. To clarify, at times we might fail to see which option is better because we lack some pertinent information, have not properly determined the criteria for comparison, or are distracted and cannot think clearly at that moment. Each of these are contingent matters which can easily be fixed by taking more time to consider the comparison at hand. In contrast, when we determine that two alternatives are on-a-par it is not a contingent matter which can be fixed, rather it is the proper way to characterise the comparative relation which holds between those options. Thus, when faced with a choice making situation between on-a-par alternatives, it is futile to ‘go back’ and reassess the alternatives so as to determine which is better. A different method is needed to choose among these options, one that does not rely on the evaluative facts which have already failed to properly guide our decision in this case.

In cases of parity Chang has argued that our method for decision making relies on our ‘normative powers’ (Chang, 2020). These powers refer to our ability as rational agents to impact the normative domain by creating new reasons for action by willing them into existence. These volitional reasons, ‘will-based’ reasons, differ from our normal reasons in that they only impact what we rationally ought to do when our normal value-based reasons, ‘given-reasons’, have run out and we are facing a choice situation involving on-a-par alternatives. In this respect this is a hierarchical method in that given-reasons will always trump our will-based reasons, but when the former run out the latter kick in.^14^

For those who are not familiar with the notion of normative powers it may seem that Chang’s suggestion employs a mystical or ad hoc solution. In fact, normative powers are much more familiar than one might think. For instance, ‘promising to do something’ can be understood as a normative power by which we create new reasons. When you promise to meet your friend for lunch you have now created new reasons for yourself to arrive at the restaurant on time, to not cancel at the last moment, and perhaps even to reject other offers that would clash with your meeting. This of course does not mean that there cannot be compelling reasons for you to cancel or miss the lunch, but if you lack such stronger reasons for not keeping your promise you would be blameworthy and open to censure for having failed to keep your promise. In similar fashion, Chang suggests that in cases of parity we determine the rational course of action not by examining the evaluative facts, for they have already been examined, but by creating new reasons through an act of commitment.^15^

Commitments are a form of normative power by which we have the ability to shape our rational identity. When confronted with on-a-par alternatives no external facts can settle the matter at hand. The way to move forward is to examine ourselves as agents and commit to one course or another and in so doing shape our own identities. For instance, if Jenny has to decide whether to continue living the life of a bachelorette or accept Jesse’s marriage proposal she may come to the conclusion that both options are good but for different reasons making neither better than the other. Similarly, Jenny can rule out the option that these two alternatives are of equal value by noticing that it would be inappropriate to base her decision on some arbitrary method such as flipping a coin. But rather than being stumped as to what is the rational thing to do, Jenny ought to embrace the idea that her alternatives are on-a-par and that the thing to do is to commit to one of these options and by doing so make it the rational choice. Jenny is in the wonderfully fortunate position of being able to actively shape her identity, she is not forced to be a slave to her reasons. In similar fashion, this same method can also be used at the communal or group level through acts of ‘self-governance’. Looking back to our nuclear reactor example, if a committee or society had to choose one of these reactors for development and they concluded that the two reactors are on-a-par, what they need to do is commit to one of these courses of action. For instance, they could decide to ‘will’ the fact that “MSR scores higher in terms of sustainability” or that “MSR promotes the group’s green identity” to be a ‘will-based’ reason for choosing MSR over HTR-PM. In willing this reason into existence, by committing to this green identity, they now make it that MSR is the rational option to choose.

Before showing how parity can be implemented in the VSD framework and the three methods for value conflict resolution, it is important to address two potential objections. First, if I have the normative power to make some option the rational choice, can’t I just justify any course of action whatsoever? This is an immediate concern but should not worry us as we have already stressed that will-based reasons are subordinate to given-reasons meaning that any option which is ruled out by some given-reason cannot then be justified by our willing a new reason in its favor.

Second, on the individual level it is clear that I, as the decision maker, need to commit towards a specific course of action. But once we move to the group level, whose commitments should be taken into consideration?^16^ This is a difficult question which has yet to be properly addressed in the parity literature. Nevertheless this issue can be at least partially answered. Chang for instance has suggested that there is a close link between collective or group commitments and theories of deliberative democracy (Chang, 2009, 155–157).^17^ Following Chang’s suggestion I believe that a general rule of thumb is that there ought to be a correlation between those who are impacted by the design choice and those who make the relevant commitment. For instance, consider two possible designs for a social media newsfeed algorithm (Bozdag & Poel, 2013). One would prioritise diversity of opinions and would actively expose users to opinions which differ from their own. Alternatively, the second design would favor posts which are most likely to be in line with the users’ own point of view. It seems reasonable to assume that these two designs could be on-a-par, one is favored by the value of diversity of opinion, the other is favoured by values such as autonomy or self determination, and overall neither is clearly better. To determine what is the rational thing to do it was suggested that a commitment needs to be made towards one of these options. One possible way forward is for the design team itself or the social media platform to make that commitment and stand behind one of these designs—‘We at social media company X are committed to diversity of opinions and so create a reason to favor this algorithm’. Alternatively, if we determine that the algorithm is likely to have an impact on the lives of the social media platform users, we might opt for allowing each user to make this commitment on her own by needing to actively choose which algorithm applies. Finally, if we conclude that the design of the algorithm may eventually impact society at large we may decide that the right body which needs to commit is the populace itself or the government. This issue clearly needs to be further developed but hopefully this gives some indication as to how to approach this issue.

Turning back now to the three methods discussed in the previous section, it can now be shown how parity neatly compliments and develops each method. Starting with ‘Value Dams and Flows’ our main concern was that the method may lead designers to commit the naturalistic fallacy of confusing the preferences of the stakeholders with what is normatively speaking the rational thing to do. Parity can provide us with a way to partially alleviate this concern. Notice that when we are forced to select among incommensurable alternatives which are on-a-par, each option has already been deemed to be acceptable and so there is no such thing as a wrong choice. Turning to the stakeholders in such cases and following their preferences can be understood merely as a method for revealing the group commitment in the face of parity. As such, by incorporating parity into the ‘Value Dams and Flows’ method we can pinpoint those instances in which a rational choice can be made only by eliciting input from the relevant stakeholders.

As to the substantive ethical theory method, parity would provide us with a way to approach and resolve conflicts between the basic values of the chosen ethical theory. For instance, van Wynsberghe’s CCVSD picks out four fundamental values: attentiveness, responsibility, competence, and responsiveness. These values are meant to act both as the basis for our evaluative analysis and as the guiding light for our design. But as was pointed out above, when these fundamental values come into conflict we lack a method for resolving such cases. Parity provides the missing link. One course of action is favored by one fundamental value, and a contrasting course of action is favored by a different fundamental value, and neither option is better overall, then we know that the rational thing to do is to make a commitment and then act in accordance.

Lastly, incorporating parity into the ‘Values Hierarchies' method should allow it to overcome the difficulties mentioned in the previous section. Recall that it was shown that each of the method’s three key actions—Specification, Respecification, and Innovation—cannot be properly conducted in the face of conflicts between incommensurable values. Parity would provide a solution in all three instances. First, parity could describe the relation that holds between various specifications and explain how it is that they can all be legitimate and acceptable. Second, when one specification leads to an unacceptable outcome this can either be understood as a value based reason for why that specification was not actually acceptable, or as the downstream effect of our commitments. In the former case we should respecify from amongst the group of remaining acceptable specifications. In the latter case we should either reconsider our commitment or accept the outcome as part of the cost we need to pay. This would be akin to a newly married woman accepting that she is no longer a bachelorette and that even though staying as a bachelorette or getting married were on-a-par options, once that decision has been made she must accept the new duties and responsibilities that come with being married. Lastly, parity would help guide designers to choose between possible innovation paths when no single pareto optimal path can be detected.

I end this paper with a short comment on how accounting for parity should impact the general framework of VSD. With regards to the investigation phase a greater emphasis needs to be put on ascertaining whether the value conflicts which are identified stem from the incommensurability of those values. In terms of stakeholder analysis, it now becomes even more important to anticipate the impact that the design is expected to have on the various stakeholders. This is a crucial step if we wish to identify who is in position to make the necessary commitments for determining how to proceed in the face of conflicts between incommensurable values.

## Conclusion

In this paper I argue that the phenomenon of incommensurable values poses a difficulty to the VSD framework and to the various theories for value conflict resolution. I then suggest that the evaluative relation of parity and its accompanying rational decision theory can improve the existing methods and further develop the general framework of VSD. Briefly put, by accepting parity as a fourth value relation we are accepting that rational decisions are not merely a function of the facts of the case, but at times are actively determined by us as active agents and designers of our normative realm. There is of course more work to be done in order to better understand the exact application of parity in the fields of responsible innovation and VSD but it is my hope that this paper is a good starting point for future research.

## Footnotes

1 For more on this see Van de Poel (2015, 2017), and Van den Hoven (2012).

2 I thank an anonymous referee for stressing this point.

3 For more on this debate see Jacobs (2020), Jacobs and Huldtgren (2021), and Cenci and Cawthorne (2020).

4 Incommensurability can be applied both to values and to value bearers. That is, abstract values can be incommensurable with respect to some third value, options in a choice situation can be incommensurable in terms of the criteria of choice, and objects can be incommensurable in terms of some particular value.

5 For a detailed discussion of incommensurability and how it differs from incomparability see Chang (1997, 2015, 2016).

6 Such arguments can be found in (De Sousa, 1974; Raz, 1988; Sinnott-Armstrong, 1985).

7 See (Chang, 2001; Parfit, 1986).

8 See Chang (2001) chapter 5 for a detailed discussion of this argument.

9 For more on this critique see Manders-Huit (2011).

10 For a detailed explanation and defence of parity see Chang (2002). For what I take to be unsuccessful objections see Wasserman (2004), Hsieh (2005) and Elson (2014).

11 For a more detailed discussion see (Chang, 2015, 16).

12 For an example of an MCDM theory and a review of its literature see Rezaei (2016).

13 See Schwartz (2012) I thank an anonymous referee for suggesting this literature.

14 See Chang (2009, 2013a).

15 For a discussion on the notion of commitments see Chang (2013b).

16 For a relevant discussion on public decision making and its relation to scientific knowledge see Martin and Richards (1995).

17 For a case study on value conflict resolution through deliberative mechanisms see Bail et al. (2020). Thank you to an anonymous referee for referring this article to me.
